# *Chlamydia trachomatis* Infections in Female Soldiers, Israel

**DOI:** 10.3201/eid0910.030100

**Published:** 2003-10

**Authors:** Ellen S. Bamberger, Efraim Siegler, Einat Makler-Shiran, Mihir V. Patel, Jordan M. Steinberg, Rosa Gershtein, Isaac Srugo

**Affiliations:** * B’nai Zion Medical Center, Haifa, Israel; † Rambam Medical Center, Haifa Israel; ‡ Albert Einstein College of Medicine, Bronx Burough of New York, New York, USA; 1 Dr. Bamberger and Dr. Siegler contributed equally.

## Abstract

We examined the prevalence of *Chlamydia trachomatis* infection in Israeli female soldiers. The prevalence was 3.2% among soldiers seeking medical care; rural residence was identified as a significant risk factor. Nevertheless, given the study design, recommending broad-scale screening of Israeli female soldiers may be premature.^2^

Recent studies from the United States and Europe report that the prevalence of *Chlamydia trachomatis* ranges from 5% to 20% in sexually active persons (*1*,*2*). Among women, the consequences of the disease include pelvic inflammatory disease, ectopic pregnancy, and infertility, sequelae often accompanied by a substantial economic impact (*3*). Most importantly, for up to 80% of infected women, infection is asymptomatic, resulting in failure to seek timely medical care and the exacerbation of such sequelae (*4*). Consequently, screening programs have been recommended to reduce infection, transmission, and disease consequences (*4*,*5*). However, before such programs are universally advocated, since the cost-effectiveness of such control programs is at least partially contingent upon disease prevalence (*5*), more data are required to assess the extent to which such prevalence rates may indeed be generalizable to other countries and populations.

Epidemiologic research indicates that prevalence rates of *C. trachomatis* vary, with a number of risk factors accounting for a substantial portion of this variance (*4*). For example, in the landmark study by Gaydos et al., significant risk factors for the prevalence of this infection among American female military recruits included age (women <25 years of age were at higher risk), vaginal intercourse, lack of condom use, and multiple sexual partners (*4*). Given that sexual practices such as condom use and multiple sex partners are likely to vary from country to country, we designed a study to assess the prevalence of *C. trachomatis* in a population of Israeli women, namely female soldiers, who, according to previous research, had a heightened risk of having the disease because of their age and sexual activity (*4*–*6*).

## The Study

Whereas Gaydos et al. invited all female army recruits undergoing a physical examination at a reception base over the course of 2-year period to participate in their study, our sample consisted of Israeli women actively seeking medical care. Specifically, participants in the current study were women serving in the Israel Defense Forces, who, over a 12-month period attended a women’s health clinic for a routine gynecologic examination, treatment of genitourinary symptoms, or contraceptive counseling. The clinic was associated with a major tertiary hospital in northern Israel. Of the 800 women we asked, 708 volunteered to participate, for a response rate of 88.5%. Participants were between the ages of 18 and 45 (median age 19, mean age 20). Most participants (77%) were born in Israel.

After signing an informed consent form, participants were asked to complete a short questionnaire (including demographic and sexual history items) and provide a urine specimen. We performed the polymerase chain reaction (PCR) of urine specimens to test for *C. trachomatis* and *Neisseria gonorrhoeae* (Roche Amplicor, Branchburg, NJ). The PCR results and questionnaire data were coded as dichotomous variables and examined on the basis of both univariate (chi-square and Fisher exact test) and multivariate (logistic regression) analysis.

Of the 708 participants, 23 (3.25%) tested positive for *C. trachomatis,* and 8 (1%) tested positive for *N. gonorrhoeae.* These rates are substantially lower than the 9.2% prevalence rate for *C. trachomatis* reported by Gaydos in the U. S. military study (*4*). The median and average age of those affected was 20. Most of those testing positive reported 1) having had sex in the past 3 months (86%), 2) having had more than one partner (45%) or having had a partner who had had more than one partner (57%), or 3) never having used a condom (70%) (Table). Nevertheless, none of these factors, identified by Gaydos as significant risk factors in the American sample, was found to be a significant predictor of *C. trachomatis* infection in the Israeli soldiers. Similarly, while most of those testing positive were born in Israel (81%), country of origin was not a significant predictor of infection. Neither were gynecologic symptoms found to significantly distinguish between *C. trachomatis–*positive and –negative soldiers. Indeed, the only factor that we found to significantly distinguish between *Chlamydia-*positive and -negative persons was urban versus rural residence. Specifically, rural residents were significantly more likely to be positive for *C. trachomatis* than urban residents (chi square=3.86, p<0.05). Similarly, in a logistic regression analysis, only rural residence was significantly associated with *Chlamydia* infection (p=0.043) (Table).

**Table:** Risk factors for *Chlamydia trachomatis* in female Israeli soldiers

Risk factor	Presence in soldiers who were		p value
	Positive for *C. trachomatis/*no. tested (%)	Negative for *C. trachomatis*/no. tested (%)	
Sex in the past 3 months	19/22 (86)	585/629 (93)	0.999
>1 partner	9/20 (45)	241/636 (38)	0.519
Partner having >1 partner	8/14 (57)	181/518 (35)	0.093
Never used a condom	14/20 (70)	442/628 (70)	0.99
Country of origin			
Israel	17/22 (81)	525/685 (77)	0.95
Other	5/22 (19)	160/685 (23)	0.805
Dysuria	4/21 (19)	136/639 (21)	0.316
Vaginal discharge	11/21 (52)	263/635 (41)	
Location of residence			
Urban	5/21 (24)	310/682 (45)	0.049
Rural	16/21 (76)	372/682 (55)	

## Conclusions

The relatively low rate of *C. trachomatis* prevalence was somewhat surprising for several reasons. First, since 1994, when *Chlamydia* infection became a nationally notifiable infectious disease in Israel, the number of cases reported annually has risen 10-fold. Secondly, since 2000, 14% of symptomatic soldiers attending the sexually transmitted disease clinic of another major medical center in northern Israel were diagnosed with *Chlamydia* infection. Finally, while Gaydos et al. found a 9.2% prevalence rate in a sample of soldiers reporting for a general physical examination, our sampling strategy focused on women who had a high probability of being sexually active (i.e., seeking contraceptives) and consequently, according to the previous literature, were at higher risk of contracting asymptomatic *C. trachomatis* infection (*5*).

However, oversampling those most concerned with their health (i.e., “the worried well”) may partially explain the lower than expected prevalence rate. Indeed, given the risk for a self-selection bias inherent in such a sampling strategy, one important limitation of the current study is that the sample population may not be entirely representative of the entire population of female soldiers in the Israeli Defense Forces. A second limitation is that the Roche PCR kit used in this study was not cleared by the Food and Drug Administration for testing female urine samples for *N. gonorrhoeae* because of its low sensitivity. Consequently, the actual prevalence of gonorrhea may in fact be higher than reported above.

Given the nearly universal draft of young Jewish females in Israel, however, our results may provide a strong indication of the prevalence of *C. trachomatis* in young, sexually active Jewish women in that country. Despite the relatively low rate of prevalence, based on a number of cost-efficacy studies (*6*,*7*) and consistent with the recommendations of the Centers for Disease Control and Prevention (*5*), focused screening of asymptomatic, sexually active female soldiers, by virtue of their age, may be a cost-effective mode for identifying those with *C. trachomatis* infection in Israel. Identifying gonorrhea in 1% of the women tested shows an important secondary benefit to such screening. Nevertheless, before recommending the adoption of a broader, more focused screening program of female soldiers, whether symptomatic or not, we recommend collecting additional data with a more randomized sampling strategy similar to that adopted by Gaydos et al. (*4*). Were an equivalent or higher prevalence rate to be found in a random sample of female soldiers during their service, then, according to recent estimates of cost-effectiveness (*7*,*8*), such a screening program would be worthy of consideration.
